# Chicken meal is not an appropriate reference protein for estimating protein quality of ingredients used in extruded diets intended for dogs

**DOI:** 10.1093/jas/skae265

**Published:** 2024-09-10

**Authors:** Michelina Crosbie, James R Templeman, Julia G Pezzali, Glenda Courtney-Martin, Crystal L Levesque, Leslie Hancock, Preston R Buff, Daniel A Columbus, Adronie Verbrugghe, Anna K Shoveller

**Affiliations:** Department of Animal Biosciences, University of Guelph, Guelph, ON, Canada N1G 2W1 (at the time of the trial); Department of Animal Biosciences, University of Guelph, Guelph, ON, Canada N1G 2W1 (at the time of the trial); Department of Animal Biosciences, University of Guelph, Guelph, ON, Canada N1G 2W1 (at the time of the trial); Department of Nutritional Sciences, University of Toronto, Toronto, ON, Canada M5S 1A8; Department of Animal Sciences, South Dakota State University, Brookings, SD 57007, USA; The J.M. Smucker Co., Orrville, OH 44667-0280, USA (at the time of the trial); The J.M. Smucker Co., Orrville, OH 44667-0280, USA (at the time of the trial); Department of Animal and Poultry Science, University of Saskatchewan, Saskatoon, SK, Canada S7N 5A8; Prairie Swine Centre, Inc., Saskatoon, SK, Canada S7K 3J4; Department of Clinical Studies, Ontario Veterinary College, University of Guelph, Guelph, ON, Canada N1G 2W1; Department of Animal Biosciences, University of Guelph, Guelph, ON, Canada N1G 2W1 (at the time of the trial)

**Keywords:** canine nutrition, indicator amino acid oxidation, methionine, metabolic availability, peas

## Abstract

The indicator amino acid oxidation (**IAAO**) method has been used to determine metabolic availability (**MA**) of amino acids in feedstuffs for pigs, humans, and preliminarily for cats. Peas are a commonly used protein source in grain-free extruded dog diets. However, peas have a poor sulfur amino acid (**AA**) ratio (methionine [**Met**]:cysteine) with Met being the first limiting AA. Furthermore, little is known about the MA of Met in peas fed to dogs. Therefore, our objective was to compare the MA of Met in peas to chicken meal (**CM**), as a gold-standard reference protein. The study was done as a replicated 5 × 5 complete Latin square design. Ten neutered male mixed-breed dogs (1.5 years old; 26.0 kg ± 2.4 kg body weight; **BW**) fed to maintain ideal BW received all dietary treatments: **BAS**: lamb-based diet (deboned lamb and lamb meal) providing Met at 50% of its requirement (0.27 g/100g dry matter [DM]), **CHK**: CM and lamb-based diet, and **PEA**: ground dried pea and lamb-based diet both providing Met at 68% of its requirement (0.35 and 0.37 g/100g DM, respectively). Two other treatments were created by blending BAS with PEA (**BAP**) and the BAS with CHK (**BAC**) to create diets with Met at 59% of requirement (0.32 and 0.31 g/100g DM, respectively). This resulted in three graded levels of Met for both CM and peas to allow for a slope-ratio assay approach to quantify MA with the BAS diet as the common first point. All other AAs were provided to meet at least 120% of the AAFCO recommendations for adult dogs. The BAS diet, with supplemental DL-Met, was fed for a 2-wk wash-in period. After 2 d of diet adaptation IAAO was performed. Dogs were fed 13 small meals where meal 6 contained a priming dose (9.4 mg/kg BW) of L-[1-^13^C]-phenylalanine (**Phe**; 99%) as well as a constant dose (2.4 mg/kg BW) in meals 6-13. Breath samples were collected and enrichment of ^13^CO_2_ was measured using isotope-ratio mass spectrometry to calculate the rate of Phe oxidation (F^13^CO_2_ umol/kg BW/h). Oxidation was analyzed via SAS using PROC GLIMMIX with dog and period as random effects, and diet, %Met, and their interaction as fixed effects. Unexpectedly, the slope of Phe oxidation, in response to increasing Met intake, from CM was 31% of that of peas, indicating a lower MA for Met in CM as compared to peas. This finding may be due to damage of AAs during rendering. At this time, CM in extruded diets is not an acceptable reference protein to determine MA of AAs in dogs, and the MA of Met from peas cannot be confidently assessed.

## Teaser

Protein quality of ingredients intended for use in extruded dog diets cannot be determined using chicken meal as a reference protein using the indicator amino acid oxidation technique.

## Introduction

Peas are a commonly used protein source in grain-free extruded dog diets (Plantz, 2017). However, peas have a low sulfur amino acid (**SAA**) ratio (methionine [**Met**]:cysteine [**Cys**]) with Met being the first limiting amino acid (**AA**; Mansilla et al., 2019). Methionine is considered together with Cys for the total SAA requirement in mammals (Mansilla et al., 2019). Sulfur amino acid concentrations are low in legume proteins (i.e., soybean meal, ~48% crude protein, ~1.4% Met and ~1.5% Cys as-fed) (NRC, 2006). Further, the Met:Cys in many legumes such as peas is also low (Met:Cys = ≤ 1:1; Olsen et al., 2020). This has the potential to limit the amount of Met available as a methyl donor and as a precursor for Cys, taurine, and pyruvate synthesis when other dietary methyl donors are also limiting in the diet (Robinson et al., 2016). Additionally, extended feeding of a SAA-deficient diet in dogs will result in decreased body protein synthesis and food intake, as well as a negative nitrogen balance (Kanakubo et al., 2015). Previously, nutritionally mediated dilated cardiomyopathy (**DCM**) in dogs has been related to poor Met bioavailability in lamb and rice diets and also to increased soybean provision in grain-free dog foods (Fascetti et al., 2003; Sanderson, 2006; FDA, 2018). This led to the identification of the sufficiency of bioavailable Met and Cys as potential variables in the purported, but not substantiated, increase in DCM cases reported by the FDA beginning in 2018 (Fascetti et al., 2003; FDA, 2018).

Trends in the pet food industry over the last decade include formulating higher total protein content without the addition of supplemental indispensable AAs, which may result in a much lower indispensable AA to dispensable AA ratio and lead to dietary AA imbalances (Buff et al., 2014). As previously mentioned, peas have low Met:Cys and with the advent of limited ingredient diets and clean label requirements, the inclusion of peas to obtain high dietary protein suggests the provision of SAA is likely imbalanced (Kanakubo et al., 2015). Compared to chicken meal (**CM**) peas also contain approximately 135% more total dietary fiber which is known to reduce ileal AA digestibility compared to animal proteins, however, limited research exists on how differing fiber contents may impact AA bioavailability (Bednar et al., 2000; Oba et al., 2019; Reilly et al., 2020; Quilliam et al., 2021; Cargo-Froom et al., 2023a). Further, heat treatment of field peas during extrusion may also reduce the bioavailability of Met and SAA contents (Cabuk et al., 2018).

The indicator amino acid oxidation (**IAAO**) technique has been used to determine metabolic availability (**MA**) of AAs in feedstuffs in pigs, humans, and preliminarily in cats (Moehn et al., 2005; Fakiha et al., 2020; Pezzali et al., 2023). The MA of an AA in an ingredient is the portion of that AA that is both digestible and available for protein synthesis (Moehn et al., 2005). Therefore, knowing the MA of an AA in an ingredient can ensure that sufficient quantities of bioavailable AAs are provided in animal diets containing that ingredient. Protein synthesis is inversely proportional to oxidation so the IAAO technique to determine MA is based on the principle that when one indispensable AA is limiting, all other AAs will be oxidized (Moehn et al., 2005). By feeding a diet with graded levels of the limiting AA (test AA; e.g., Met) below the requirement, oxidation in response to the provision of Met can be measured by feeding a labeled indicator AA (e.g., L-[1-^13^C]-phenylalanine). Metabolic availability of the test AA in an ingredient can then be calculated in comparison to a reference diet, which is assumed to contain the test AA in its 100% bioavailable form (Moehn et al., 2005). Proper diet design is crucial when conducting MA studies; 1) all diets must be both isoenergetic and isonitrogenous to ensure this does not influence the uptake of the test AA, 2) the test AA must be fed at a minimum of three graded levels in both the reference diets and the test diets to allow for a slope-ratio approach, 3) dietary concentrations of the unlabeled indicator AA and any downstream AAs (e.g., phenylalanine and tyrosine) must be provided in excess, and 5) all other indispensable AAs must be provided above their dietary requirement to ensure oxidation of the indicator AA is only influenced by the provision of the test AA (Moehn et al., 2005).

Metabolic availability studies in swine and cats primarily use a mash-type reference diet where all AAs are provided as crystalline AA, which is considered to be 100% digestible and bioavailable (Moehn et al., 2005; Fakiha et al., 2020). These mash-type diets are typically used to minimize heat damage of AAs which may impact their MA (Moehn et al., 2005). However, over 60% of dogs consume extruded kibble as their primary diet which emphasizes the need to conduct canine MA studies using extruded diets to capture differences in AA availabilities that may be impacted due to processing (Dodd et al., 2020; Geary et al., 2023). Chicken meal is a gold-standard protein source used commonly in the pet food industry and has been well-researched in terms of its AA composition and protein quality, leading to its use in extruded dog food formulations for decades (Bednar et al., 2000; Faber et al., 2010; Oba et al., 2019). Therefore, CM was selected as the reference protein instead of a crystalline AA mash-type reference diet for this study.

Currently, little is known about the MA of Met in peas in dogs. Research into the MA of Met from field peas is necessary to determine if diets with high pea inclusion as the major protein source may or may not provide sufficient concentrations of MA Met to meet the dogs’ physiological requirements. Therefore, the objective of this study was to compare the MA of Met in peas to CM, as a gold-standard reference protein. It was hypothesized that peas would have a lower MA of Met compared to CM due to the low Met:Cys ratio and higher total dietary fiber content of peas.

## Materials and Methods

The experimental protocol and study design were reviewed and approved by the University of Guelph Animal Care Committee (AUP# 4531). Handling and care of the animals were in accordance with the Canadian Council on Animal Care Guidelines (CCAC, 2020).

### Animals, housing, and acclimation to oxidation chambers

Ten neutered male mixed-breed hound dogs (1.5 years old; 26.0 kg ± 2.4 kg bodyweight; **BW**) were obtained from Marshall Bioresources (North Rose, New York, United States of America). All dogs resided at the University of Guelph Central Animal Facility (Guelph, Ontario, Canada). Eight dogs were single-housed, and one pair of dogs was housed together in kennels (3.7 m length, 2.0 m width, and 2.0 m tall) with nose-to-nose and visual contact. The room was maintained under constant environmental conditions with a mean temperature of 20.8 °C, a mean relative humidity of 59.0%, and a 12:12 (L:D) h lighting schedule. Within their kennels dogs had unlimited access to rubber and nylon toys and water. All dogs received 20 min of supervised outdoor walks 6 d/wk unless the weather was poor, in which case dogs were walked indoors.

Prior to the trial, all dogs were adapted to eating in crates (0.9 m length, 0.6 m width, and 0.6 m tall) over a 4-wk period and then to being fed their full daily ration in crates throughout the study. Once dogs were adapted to eating in crates, the dogs were walked 10 min from the Central Animal Facility to a separate testing site located within the Animal Science and Nutrition building at the University of Guelph (Guelph, ON). Each dog was placed in the oxidation chamber to simulate the testing environment that contained crates similar to those used in training to maintain dog comfort and familiarity. Initially, dogs were placed in the oxidation chamber for a 1-h period and fed two meals (each representing one-half of their total daily ration). Over a 4-wk period, dogs were gradually adapted to being fed in the oxidation chamber every 30 min over a 4-h period. Afterward, the dogs were returned to the Central Animal Facility. This acclimation protocol (Shoveller et al., 2017) was used to minimize potential stress to the dog that would affect whole-body metabolism and confound CO_2_ production.

### Diets and study design

Three extruded kibble diets were formulated to determine MA of Met in peas (Table 1). These included a lamb-based diet containing deboned lamb and lamb meal (**BAS**) providing Met at 50% of its requirement for Labrador Retrievers (0.27% Met dry matter (**DM**), Table 2; Mansilla et al., 2020), a CM and lamb-based diet (**CHK**), and a ground dried pea and lamb-based diet (**PEA**) both providing Met at 68% of its requirement (0.35% and 0.37% Met DM, respectively, Table 2; Mansilla et al., 2020). All protein-containing ingredients were added at the expense of cornstarch to make diets isoenergetic and to meet pre-determined Met content targets, but not crude protein contents (Table 1). All other nutritional contents (except for fiber) were formulated to be held constant across all three diets. Fiber was not held constant due to the inherently high fiber content of peas and is a variable that may alter digestibility and bioavailability (Silvio et al., 2000). Barley was provided at the same inclusion level across all diets and lamb meal and deboned lamb were provided at similar inclusion levels across all diets. Lamb meal and deboned lamb were provided in the BAS diet at inclusion levels of 15.87% and 10%, respectively, (vs. 15% and 9.5% lamb meal and deboned lamb in the CHK and PEA diets, respectively) in order to provide Met at 50% of the requirement (0.27% Met DM; Mansilla et al., 2020). Barley, lamb meal, and deboned lamb were used in all diets due to their inherently low Met content and to allow for changes in Met MA to be reflective of the inclusion level of the CM reference protein and peas (Moehn et al., 2005). The targeted processing parameter for all three diets was to have a similar wet bulk density out of the extruder to ensure consistent cooking, therefore, all diets were extruded using the same processing parameters aside from extruder motor load (BAS: 83.6 A, CHK: 77.6 A, PEA: 62.5 A). Extruder motor load is a function of the extruder motor itself and the ingredient composition of the diet, so these differences were expected (Phillips, 1989). After formulation and production of test diets, it was determined through nutrient analysis that the provision of Met in the CHK diet was greater than that of the Met provision in the PEA diet. To correct for this effect, the CHK diet used in this study was created by blending 80% of the CHK diet with 20% of the BAS diet to achieve a similar level of Met provision to the PEA diet (0.35% DM and 0.37% Met DM, respectively; Table 2). Phenylalanine (**Phe**), tyrosine (**Tyr**), cysteine (**Cys**), and tryptophan (**Trp**) were added as free AA to meet additional requirements for determining MA of AA in ingredients using the IAAO technique (Moehn et al., 2005). These AAs were provided on an equimolar basis, in a solution of distilled water heated to a maximum of 50 °C to ensure free AAs were completely dissolved and then top-dressed on the diet prior to feeding to minimize heat damage to these AAs. Phenylalanine content of all diets was matched to the PEA diet which contained over 200% of the Association of American Feed Control Officials (**AAFCO**) recommendations for adult dogs (2023) on a DM basis (1.06% vs. 0.45%, respectively; Table 2) and Tyr was provided at 110% of the Phe provision on a DM basis across all diets to ensure Phe would be shunted to either protein synthesis or oxidation and not to synthesize Tyr (Shiman and Gray, 1998). Before the study began it was found that all bags of the PEA diet had molded and therefore had to be remade (Crosbie et al., 2024). Unfortunately, updated analytical values for all AA from this second production run of the PEA diet were unavailable until after the study had already begun due to extenuating circumstances. Therefore, the predicted diet AA contents of the PEA diet from the formulation software were used to determine free AA dosing in the PEA diet. The predicted AA contents of the PEA diet were accurate predictors of the analytically determined nutrient contents received from the first production run of the PEA diet and the formulation was identical between runs (Table 1). Therefore, these predicted diet AA contents were used to determine free AA dosing in the PEA diet confidently. This resulted in the PEA diet having a slightly greater Tyr provision compared to the BAS and CHK diets (1.12% DM vs. 1.08% DM, respectively). However, as the Tyr provision was still greater than 110% of the Phe provision this was not considered biologically significant. Cysteine content of all diets was also matched to the PEA diet as this was over 50% of the AAFCO (2023) total SAA recommendations on a DM basis (Table 2). All other AAs, including Trp, were provided with at least 120% of the AAFCO recommendations for adult dogs on a DM basis to ensure protein synthesis would not be limited by another AA (AAFCO, 2023). Diets were made isonitrogenous via supplementation with free alanine (**Ala**) in solution to match the crude protein contents of all diets to the PEA diet, which had the highest crude protein level (25.37% crude protein on a DM basis; Table 2) and prepared using the same standards mentioned above. Two other treatments were created by blending the BAS and PEA diet (50% BAS and 50% PEA; **BAP**) and BAS with CHK (60% BAS and 40% CHK; **BAC**) to create diets with Met at 59% of its requirement (0.32% and 0.31% Met DM, respectively, Table 2; Mansilla et al., 2020). This resulted in three graded levels of Met for both CM and peas to allow for a slope-ratio assay approach with the BAS diet as the common first point (Moehn et al., 2005).

**Table 1.** Ingredient formulation of basal (BAS), chicken (CHK), and pea (PEA) diets used to determine the metabolic availability of Met in peas on as-fed (%) basis^1^

	Diet		
Ingredient, %	BAS	CHK	PEA
Peas, ground dried	—	—	44.14
Chicken meal	—	7.11	—
Lamb meal	15.87	15.00	15.00
Lamb, deboned	10.00	9.50	9.50
Corn starch	42.49	36.90	—
Barley, pearled	20.00	20.00	20.00
Chicken fat	5.61	5.58	6.77
Beet pulp	3.00	3.00	2.39
Animal digest	1.00	1.00	1.00
Potassium chloride	0.80	0.71	0.05
Salt	0.50	0.50	0.50
Vitamin mix	0.26	0.26	0.26
Mineral mix	0.25	0.23	0.21
Choline chloride	0.16	0.14	0.13
Naturox, dry	0.03	0.03	0.03
Naturox, liquid	0.03	0.03	0.03

^1^Formulation of all 3 test diets are as formulated and do not reflect any blending of diets.

BAS = Basal diet containing lamb as the primary protein source; CHK = Chicken meal diet containing chicken meal and lamb as the primary protein sources; PEA = Pea diet containing peas and lamb as the primary protein sources.

**Table 2.** Analyzed and calculated nutrient and amino acid contents of the basal reference diet with 50% of the Met requirement (BAS), the basal + chicken (BAC) and basal + pea (BAP) diets with 59% of the Met requirement, and the chicken (CHK) and pea (PEA) diets with 68% of the Met requirement after blending and supplementation used to determine the metabolic availability of Met in peas on a DM-basis (unless specified)

	Diet^1^									
Item	BAS		BAC		BAP		CHK		PEA	
% Met req.^2^	50		59		59		68		68	
ME, kcal/kg as-fed^3^	3,433		3,445		3,488		3,437		3,498	
Dry Matter, %	91.62		90.08		91.74		90.85		91.86	
Crude protein, %^4^	25.37 (16.26)		25.37 (18.23)		25.37 (20.70)		25.37 (20.20)		25.37	
Crude fat, %	10.8		11.4		12.8		12.0		14.8	
Crude fiber, %	1.6		1.6		3.2		1.6		4.8	
Total dietary fiber, % as-fed	6.62		5.93		9.42		5.24		12.21	
Soluble dietary fiber, % as-fed	1.76		1.92		1.89		2.09		2.01	
Insoluble dietary fiber, % as-fed	4.86		4.00		7.53		3.15		10.20	
NFE, g/100g as-fed^5^	59.2		52.6		47.9		48.9		43.8	
Ash, %	6.8		7.1		7.2		7.3		7.4	
Indispensable AA, %	Diet^6^	Free^7^	Diet	Free	Diet	Free	Diet	Free	Diet^8^	Free
Arg	1.01		1.16		1.40		1.31		1.78	
His	0.29		0.34		0.41		0.39		0.53	
Ile	0.51		0.60		0.70		0.68		0.89	
Leu	1.09		1.23		1.44		1.37		1.78	
Lys	0.77		0.92		1.10		1.06		1.42	
Met	0.27		0.31		0.32		0.35		0.37	
Phe	0.61	0.46	0.69	0.38	0.84	0.23	0.76	0.31	1.06	
Thr	0.60		0.67		0.76		0.74		0.91	
Trp	0.15	0.04	0.17	0.02	0.19	0.02	0.19		0.22	
Val	0.75		0.84		0.97		0.94		1.18	
Dispensable AA, %	Diet^6^	Free^7^	Diet	Free	Diet	Free	Diet	Free	Diet^8^	Free
Ala	1.00	8.77	1.12	6.75	1.22	4.39	1.24	4.97	1.43	
Asp	1.18		1.35		1.72		1.53		2.25	
Cystine^9^	0.20	0.11	0.22	0.09	0.25	0.06	0.24	0.07	0.31	
Tyr	0.47	0.71	0.53	0.65	0.62	0.58	0.58	0.60	0.78	0.44
Glu	2.26		2.52		3.04		2.77		3.82	
Gly	1.47		1.61		1.71		1.74		1.94	
Pro	1.21		1.31		1.43		1.41		1.65	
Ser	0.67		0.73		0.89		0.80		1.12	

^1^BAC diet created by blending 60% BAS diet with 40% CHK diet to hit intermediate level of Met to match the BAP diet (0.31%). BAP diet created by blending 50% BAS diet with 50% PEA diet to create intermediate level of Met (0.31%). CHK diet was created by blending 80% CHK diet with 20% BAS diet to match the Met level of the PEA diet (target = 0.35%).

^2^Methionine requirement determined as Met requirement for Labrador Retrievers (0.52 g/100 g DM; Mansilla et al., 2020).

^3^ME calculated using modified Atwater calculation.

^4^Presented as: final crude protein content after supplementation with Ala (crude protein content of the diet prior to supplementation with Ala).

^5^NFE calculated using NFE, g/100g = 100 − (moisture + Crude Protein + Crude Fat + Crude Fiber + Ash).

^6^The amount of AA (% DM) that is found in the diet as determined by analytical methods.

^7^The amount of AA (% DM) added as free-AA to ensure the same Cys, Tyr, and Phe provision across diets which was matched to the PEA diet. For Ala, this represents the amount of Ala (% DM) added to the diet in order to make diets isonitrogenous and match to the crude protein content in the PEA diet. Tryptophan was supplemented when it fell below 120% of the AAFCO recommendation for adult dogs at maintenance.

^8^To determine free-AA dosing, diet AA values for the PEA diet were predicted using formulation software, as the first run of our PEA diet went moldy before the study began and the diet had to be remade. Updated analytical AA values from the second run of the PEA diet were not available at that time due to extenuating circumstances. However, these previously predicted values were accurate predictors of nutrient analysis results received for the first run of the PEA diet and so were used confidently. The values presented here are the analytical values that were received after the study concluded.

^9^Values reported are on a cystine-basis. However, cysteine was used as the free-AA as it was easier to work with in solution. Cysteine dosage was determined relative to the mol/kg of cystine provided in the diet.

The study design was a replicated 5 × 5 complete Latin square (*n* = 10), where all dogs were randomly assigned to one of the five treatments in each experimental period, with no dog receiving the same order of treatments and ensuring that no treatment was repeated on each calorimetry day per period. During the 14-d diet adaptation period, dogs were fed the BAS diet with supplemental DL-Met provided to the AAFCO recommendation for adult dogs (0.33% DM; AAFCO, 2023) in one meal fed at 0800 h in amounts known to maintain ideal individual BW based on historical feeding records. Dogs were allowed 15 min to finish their daily meal and all dogs finished their daily ration during that time. There were five 3-d experimental periods conducted consecutively; the first 2 d were diet adaptation to the treatment diet, followed by breath collection on day 3. During the 2-d diet adaptation period typical IAAO studies require feeding animals the same amount of food in g/kg BW to ensure all dogs are adapted to receiving the same amount of dietary nitrogen (Moehn et al., 2005; Shoveller et al., 2017). However, despite all dogs in this study being of similar ideal BW, their daily energy requirement to maintain ideal BW varied from 1,110 kcal/d to 1,800 kcal/d, which meant that feeding all dogs in this way would result in some dogs undergoing weight loss and some weight gain. As weight loss and weight gain alter metabolism, it was decided that during the 2-d diet adaptation period all dogs would continue to be fed in order to maintain ideal BW, as is a principle of MA IAAO studies (Moehn et al., 2005; Shoveller et al., 2017). To control for variation that this would cause, the same quantity of each experimental diet on an energy basis was fed to dogs, on all days of the study. Additionally, all free-AA supplementation was dosed as a proportion of diet fed to ensure that all dogs received the same proportion and balance of AAs per total daily amount fed, despite these amounts being different. On IAAO breath collection days, food intake was restricted to 13 g/kg BW which varied between 100% and 57% of the historical feeding allowance known to maintain ideal BW. After completion of each IAAO, dogs that had not received their total daily feed allowance received the remainder, in order to maintain ideal BW. This feeding protocol ensured that dogs consumed all the test diets during IAAO and received equivalent isotope delivery. This 3-d feeding protocol was repeated five times until all dogs received all treatments. Blood samples (5 mL) were collected from each dog within 30 min after their last meal at the end of each IAAO day from the cephalic vein in a 10-mL sodium heparin tube (Becton Dickinson Canada Inc., Mississauga, ON) and placed on ice. Once all samples were collected, 1 mL of whole blood was separated and stored at −18 °C. The other 4 mL of blood was centrifuged at 4 °C at 15,000 × *g* for 15 min. Plasma was separated then all samples were stored in a −80 °C freezer until analysis of fed-state plasma and whole blood AA concentrations.

### IAAO study

On the day each IAAO study was conducted dogs were first weighed to determine food, isotope, and AA solution dosing then dogs were moved to individual oxidation chambers to which they had previously been acclimated to as described above. After 30 min of gas equilibration, triplicate measurements of resting volume of expired CO_2_ and O_2_ (**VCO**_**2**_; **VO**_**2**_; respectively) were taken. These measurements were taken during the fasted state due to the increased variability associated with fed-state-VCO_2_ in free-living animals. Dogs were then fed (Time 0) their corresponding food allowance divided into 13 equal small meals; the first three meals were fed every 10 minutes to induce a fed-state, and the other ten meals were fed every 25 min. The total amount of food fed during the IAAO study was based on BW measured the same morning after 22 h of fasting (13 g/kg BW). Background ^13^C enrichment was determined by collecting CO_2_ samples over three consecutive 25-min periods. The sixth meal (95 min after first feeding) contained a priming dose (9.4 mg/kg BW) and constant dose (2.4 mg/kg BW) of L-[1-^13^C]-Phe (99%; Cambridge Isotope Laboratories, Inc., Tewksbury, MA). To achieve and maintain isotopic steady state in the L-[1-^13^C]-Phe pool, the following seven meals contained constant doses (2.4 mg/kg BW) of L-[1-^13^C]-Phe for all dogs. Expired CO_2_ was collected over the last eight 25-min periods. Overall, during each IAAO study, each dog spent approximately 6.3 h inside the oxidation chamber. Additional details regarding the timeline for each IAAO study can be found in Mansilla et al. (2018).

### Sample collection and analysis

Expired CO_2_ in breath was collected using an open circuit calorimetry system that pulled fresh air into the oxidation chambers by a rotary vane vacuum pump through a series of DRIERITE-filled columns (calcium sulfate impregnated with cobalt chloride as an indicator; W. A. Hammond DRIERITE Co. Ltd) to the CO_2_ analyzer (Qubit Model S155, Qubit Systems Inc., Kingston, ON) and the gas switcher. From the gas switcher, expired breath was pushed through midget bubblers, which contained 8 mL of 1 mol/L sodium hydroxide (**NaOH**) solution. The purpose of the NaOH solution was to trap CO_2_ released by the dogs while in the oxidation chambers for the subsequent ^13^CO_2_ enrichment analysis (Shoveller et al., 2017). Breath samples were then stored in an air-tight serum tube and kept at room temperature until further analysis. Calorimetry data was collected automatically using Qubit calorimetry software (Customized Gas Exchange System and Software for Animal Respirometry; Qubit Systems Inc.). Analysis of ^13^C enrichment in breath samples was conducted at the Environmental Isotope Laboratory, University of Waterloo (200 University Ave W, Waterloo, ON). Breath samples were analyzed with a Gasbench II interfaced with a Delta V Plus mass spectrometer (Thermo Scientific, Bremen, Germany).

Free AA concentrations in plasma and whole blood were analyzed using ultraperformance liquid chromatography (**UPLC**). In short, 100 μL of plasma or whole blood was deproteinized using 100 μL of 10%-sulfosalicylic acid, vortexed, and then centrifuged at 14,000 × *g* for 5 min. The supernatant was derivatized by an ACCQTag Ultra derivatization kit (Waters Corporation, Milford, MA). Derivatized AAs were separated in a column (2.1 mm × 100 mm, particle size = 1.7 μm) maintained at 55 °C with the use of UPLC (Waters Corporation) with UV detection (260 nm). AA peak areas were compared with known standards and analyzed with Waters Empower 2 Software (Waters Corporation).

Total plasma Cys, homocysteine (**Hcys**), and glutathione (**GSH**) were analyzed with UPLC using an adapted method from Vester and Rasmussen (1991) and Pfeiffer et al. (1999). Briefly, 30 μL of the reducing agent, tris(2carboxyethyl)phosphine (Sigma-Aldrich, St. Louis, MO) in 10X phosphate buffered saline (Thermo Fisher Scientific) was added to 75 μL of plasma and 75 μL of the internal standard, N-(2-Mercaptopropionyl)glycine (Sigma-Aldrich) in 0.1 M K-borate (pH 9.5) + 2 mM EDTA. The samples were placed in the refrigerator for 30 min and then 125 μL of the derivatizing agent, 70% perchloric acid (Sigma-Aldrich), was added. Samples were allowed to sit for 10 min before being centrifuged at 13,000 × *g* for 5 min. In a light-sensitive centrifuge tube, 30 μL of supernatant was added to 60 μL of 2 M K-borate (pH 10.5) + 5 mM EDTA and 30 μL of the fluorescent thiol-specific dye, 7-fluorobenzofurazan-4-sulfonic acid ammonium salt (Sigma-Aldrich) in 0.1 M K-borate (pH 9.5) + 2 mM EDTA. Samples were then incubated in a water bath maintained at 60 °C for 60 min and then immediately put on ice for 5 min. Samples were centrifuged again at 13,000 × *g* for 5 min and then 50 μL was transferred to a UPLC vial for analysis. The derivatized thiols (1 μL injection) were separated in an Acquity UPLC BEH C18 Column (2.1 × 50 mm, 1.7 μm; Waters Corporation) that was maintained at 28 °C using UPLC with fluorescence detection at 515 nm emission and 385 nm excitation. Peak areas were compared with known standards and analyzed with Waters Empower 2 Software (Waters Corporation).

Nutrient contents of the BAS, CHK, and PEA diets were analyzed at the commercial laboratory Eurofins Microbiology Laboratories (Madison, Wisconsin, USA) for DM, crude protein, crude fat, crude fiber, total dietary fiber, soluble dietary fiber, insoluble dietary fiber, and all AA. Amino acids in the BAS, CHK, and PEA diets, except for Trp, Met, cystine, and taurine (**Tau**) were analyzed using the acid hydrolysis procedure (AOAC, 2005; 982.30). Methionine and cystine were quantified using the oxidative hydrolysis procedure (AOAC, 2005; method 994.12), Trp was analyzed using the alkaline hydrolysis procedure (AOAC, 2005; method 988.15), and Tau was analyzed using method 999.12 (AOAC, 2005). Nutrient contents of the BAC and BAP diets were determined by calculating the amount of each nutrient provided by each of the BAS and CHK or BAS and PEA diets, respectively, after blending in the proportions previously described.

### Calculations

The fraction of ^13^CO_2_ released per kg of BW per h (F^13^CO_2_ mmol/kg BW/h) was calculated using the following equation:


$$\begin{array}{l} F^{13}CO_{2}\left(mmol/kg/h\right)=\frac{\left(VCO_{2}\right)\left(APE\right)\left(44.6\right)\left(60\right)}{\left[\left(BW\right)\left(1.0\right)\left(100\right)\right]} \end{array}$$

In which VCO_2_ is the average production of CO_2_ from all breath collection days within each dog at resting in mL/min; **APE** (atom percent excess) is the average ^13^CO_2_ enrichment in an expired breath at isotopic steady state in percent; and BW is the weight of the dog in kg. The constants 44.6 (mmol/mL) and 60 (min/h) convert the VCO_2_ to micromoles per hour; the factor 100 changes APE to a fraction; and the 1.0 is the retention factor of CO_2_ in the body due to bicarbonate fixation as reported previously (Shoveller et al., 2017).

The MA of Met was calculated following the equation by Littell et al. (1997):


$$\begin{array}{l} Metabolic\;availability\;=\frac{bT}{bR} \end{array}$$

Where bT and bR were the slopes from the IAAO response (i.e., F^13^CO_2_) following graded intake of the pea test diets and reference CM diets, respectively.

Resting and fed energy expenditure (**REE**, **FEE**) were calculated based on VO_2_ and VCO_2_ using the modified Weir equation (Weir, 1949):


$$\begin{array}{l} Energy\;expenditure\;\left(kcal/d\right)\;=\;3.94\left(VO_{2}\right)+\;1.11(VCO_{2}) \end{array}$$

In which VO_2_ and VCO_2_ are the volume of oxygen consumed by the dog and the volume of carbon dioxide produced (L/d) and energy expenditure (kcal/d) was expressed in relation to metabolic BW (BW^0.75^; **mBW**) for all dogs. Resting and fed respiratory quotient (**RQ**) was calculated directly by the Qubit calorimetry software as CO_2_ production/O_2_ production. Fat and carbohydrate oxidation (g/min/kg BW) were calculated using the equations established by Frayn (1983):


$$\begin{array}{l} Carbohydrate\;oxidation\;\left(g/min/kg\;BW\right)\;=\frac{4.59\left(VCO_{2}\right)\;-3.22\left(VO_{2}\right)}{BW} \end{array}$$

$$\begin{array}{l} Fat\;oxidation\;\left(g/min/kg\;BW\right)\;=\frac{1.70\left(VCO_{2}\right)\;-1.70\left(VO_{2}\right)}{BW} \end{array}$$

In which VCO_2_ and VO_2_ are the volume of carbon dioxide produced and volume of oxygen consumed by the dog (L/min) and BW is the weight of the dog in kg.

### Statistical analysis

Statistical analyses were conducted using SAS (SAS Institute Inc., version 9.3). Plasma and whole blood AAs, BW, mBW, REE, FEE, fasted and fed RQ, fat oxidation, and carbohydrate oxidation were analyzed using the PROC GLIMMIX procedure with diet as the fixed effect with dog and period as random effects. Results from this analysis were expressed as least squares means ± SEM and means were separated using the Tukey–Kramer post-hoc test. Methionine intake was expressed as the % of Met from the estimated requirement determined by Mansilla et al. (2020) for Labrador Retrievers (0.52% DM), which were of similar size to the dogs used in this study. Regression within the analysis of variance was determined using PROC GLIMMIX to construct the regression equation for each of the dietary treatments, CM reference and PEA test diets, to obtain the slopes of the two lines. The effect of Met inclusion, the addition of Met via CM or pea inclusion, and their interaction on the variations of APE and F^13^CO_2_ were tested using PROC GLIMMIX, with dog and period as random effects. This procedure also tested whether the APE or F^13^CO_2_ slopes were significantly different from zero. F^13^CO_2_ results were expressed as a regression equation. Results were considered statistically significant *P* ≤ 0.05, a trend when 0.05 < *P* ≤ 0.10, and *P* > 0.10 was considered not significant.

## Results

All dogs remained healthy and maintained their ideal BW throughout the study. Further, all dogs consumed all meals during each 2-d adaptation period and during all IAAO sampling days prior to the corresponding breath sample collection.

### MA of Met in peas

**Figure 1.**
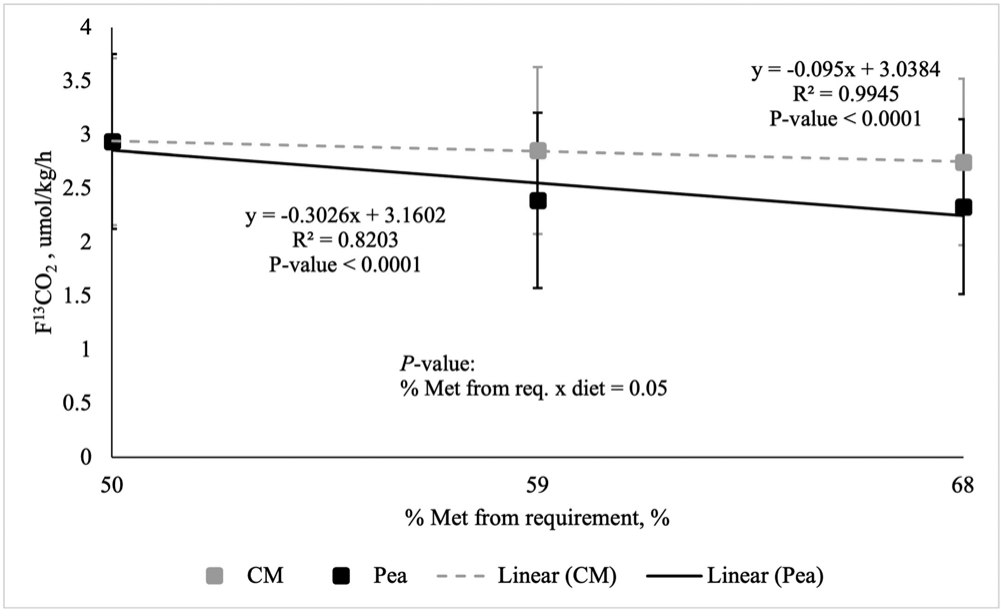
Linearity of the rate of L-[1-^13^C]-Phe oxidation (F^13^CO_2_) in response to graded intake of protein-bound Met from chicken meal (CM) and peas in adult dogs (*n* = 10). Methionine from both CM and peas was provided at both 59% and 68% of the requirement for Labrador Retrievers (0.52% DM). A lamb and barley-based BAS diet provided Met at 50% of the Met requirement for Labrador Retrievers (0.52% DM) and was used as the common first point.

The increasing concentration of Met, the different diet types, and the interaction between the two were sources of variation for F^13^CO_2_ (*P* ≤ 0.05; Figure 1). As Met intake from the reference protein (i.e., CM) increased from 50% to 68% of the requirement for Met (0.52% DM; Mansilla et al., 2020), the rate of ^13^C-Phe oxidation (F^13^CO_2_) decreased linearly (Figure 1). A negative slope of the best-fit line of −0.095 ± 0.01 (*P* < 0.0001) for the reference protein CM was determined using linear regression. As Met from the common first point increased from 50% to 68% of the requirement of Met (0.52% DM; Mansilla et al., 2020), the rate of F^13^CO_2_ from peas decreased linearly and the slope (−0.303 ± 0.01) was different from zero (*P* < 0.0001; Figure 1). However, as the rate of F^13^CO_2_ was greater for peas (more negative) than the CM reference diet, the MA of Met in peas was unable to be calculated and determined. Despite this, the MA of Met in CM can be concluded to be 31% of that of peas by dividing the slope of oxidation from CM by the slope of oxidation from peas.

### Plasma and whole blood AA

The results for plasma and whole blood AAs from blood samples taken after each IAAO sampling are presented in Table 3. There was a significant effect of diet for most of the analyzed AAs that followed a similar pattern to what was provided in each diet with the PEA and BAP diets generally providing the most AAs (and the greatest amount of AAs in plasma and whole blood) followed by the CHK, BAC, and BAS diets.

**Table 3.** Fed plasma and whole blood amino acid concentrations after indicator amino acid oxidation in adult large breed dogs (nmol/mL)

	Diet^1^						
Item	BAS	BAC	BAP	CHK	PEA	SEM^2^	*P*-value
*n*	10	10	9	10	8		
% Met req.	50	59	59	68	68		
Cys	118.5	119.7	124.6	125.2	127.5	5.27	0.173
Hcys	6.2^b^	6.2^b^	6.9^a,b^	7.0^a,b^	7.5^a^	0.66	0.003
GSH	9.8	9.1	9.9	9.1	9.0	0.62	0.589
Plasma AA							
Indispensable							
Arg	111.7^d^	116.3^d^	142.9^b^	127.7^c^	168.2^a^	3.80	<0.001
His	89.0^b^	91.5^b^	101.3^a^	96.4^a,b^	103.7^a^	3.08	<0.001
Ile	42.2^d^	46.8^c,d^	56.2^b^	50.7^b,c^	64.1^a^	1.96	<0.001
Leu	95.1^d^	100.3^c,d^	114.5^b^	106.2^b,c^	125.7^a^	4.01	<0.001
Lys	106.8^c^	112.6^c^	143.3^b^	133.0^b^	173.2^a^	8.14	<0.001
Met	34.5^b^	36.7^a,b^	37.0^a,b^	40.6^a^	38.1^a,b^	1.80	0.003
Phe	84.6^a^	81.6^a,b^	79.8^a,b^	80.7^a,b^	76.4^b^	2.93	0.004
Thr	90.9^c^	91.3^c^	114.6^b^	106.2^b^	135.7^a^	5.60	<0.001
Trp	118.1^b,c^	112.7^c^	124.4^a,b^	112.8^c^	130.8^a^	4.47	<0.001
Val	168.3^c^	175.0^b,c^	187.0^b^	182.5^b^	204.2^a^	5.63	<0.001
Dispensable							
Ala	996.4^a^	833.0^b^	577.2^c^	695.1^b,c^	362.6^d^	32.14	<0.001
Asn	37.9^c^	38.5^c^	55.5^b^	40.9^c^	67.2^a^	1.75	<0.001
Asp	5.4	4.5	5.4	6.1	4.8	0.45	0.105
Cystine	23.3	20.2	23.0	21.2	20.5	1.85	0.219
Tyr	122.5^a^	106.3^b^	99.3^b,c^	105.5^b^	92.5^c^	5.49	<0.001
Gln	871.1^a^	848.8^a,b^	855.9^a,b^	798.9^b^	863.9^a^	28.11	0.017
Glu	85.4	82.3	85.4	81.4	85.7	4.35	0.357
Gly^3^	299.6	301.4	324.3	326.2	314.8	10.15	0.015
Pro	238.1	234.7	239.8	242.6	234.3	6.73	0.774
Ser	144.5^a^	137.8^a,b^	128.0^b,c^	140.7^a,b^	115.3^c^	6.51	<0.001
Tau	80.6	82.6	86.2	93.0	80.7	7.66	0.273
Whole blood							
Indispensable							
Arg	214.3^b^	206.4^b^	224.4^b^	224.5^b^	257.3^a^	7.43	<0.001
His	123.8	119.3	123.3	127.5	129.5	4.00	0.426
Ile	67.9^b^	68.8^b^	73.4^b^	73.5^b^	85.1^a^	2.44	<0.001
Leu	139.4^b^	137.9^b^	143.5^a,b^	145.5^a,b^	160.1^a^	5.12	0.010
Lys	279.5^b^	272.0^b^	277.4^b^	297.7^a,b^	319.9^a^	9.53	0.001
Met	38.3	38.0	37.0	42.1	39.0	1.67	0.100
Phe	96.1	89.4	86.2	90.9	86.4	3.41	0.163
Thr	139.8^b^	140.8^b^	147.7^b^	152.4^a,b^	170.7^a^	5.74	0.001
Trp	50.2	46.0	48.3	46.8	51.9	2.46	0.066
Val	190.0^b^	186.6^b^	193.0^a,b^	198.8^a,b^	214.4^a^	5.93	0.013
Dispensable							
Ala	898.7^a^	743.3^b^	546.7^c^	657.3^d^	374.4^e^	23.76	<0.001
Asn	39.4^b^	37.7^b^	41.8^b^	39.1^b^	49.0^a^	2.66	<0.001
Asp	79.1	76.1	74.2	77.9	80.7	3.44	0.546
Cystine	6.6	6.1	6.5	7.0	6.6	0.34	0.164
Tyr	130.6^a^	109.5^b^	106.0^b^	112.1^b^	103.0^b^	4.23	<0.001
Gln	568.5^a^	501.4^b^	490.7^b^	507.7^a,b^	526.4^a,b^	26.62	0.017
Glu	350.1^a,b^	340.6^a,b^	334.7^b^	344.3^a,b^	374.8^a^	14.66	0.038
Gly	314.2	318.9	315.0	338.0	333.6	12.59	0.259
Pro	244.9	240.7	236.2	252.2	248.1	7.88	0.569
Ser^3^	203.9	198.3	182.7	200.6	185.2	7.78	0.037
Tau	166.2	159.5	161.3	175.2	168.6	8.20	0.292

^1^BAS = basal diet containing lamb as the primary protein source providing Met at 50% of the requirement; BAC = blend of the BAS and CHK diets providing Met at 59% of the requirement; BAP = blend of the BAS and PEA diets providing Met at 59% of the requirement; CHK = blend of the CHK and BAS diets with chicken and lamb as the primary protein sources providing Met at 68% of the requirement; PEA = PEA diet containing peas and lamb as the primary protein sources providing Met at 68% of the requirement.

^2^Greatest value for SEM.

^3^No significant differences were observed between diets when pairwise comparisons were analyzed using the Tukey–Kramer adjustment.

For the plasma AAs, Hcys concentrations were greater in the PEA diet than in the BAC and BAS diets (*P* < 0.05). Of the plasma indispensable AAs, arginine (**Arg**) was greatest in the PEA diet followed by the BAP diet, CHK diet, and BAC and BAS diets (*P* < 0.05). Plasma histidine (**His**) was greater in the PEA and BAP diets than in the BAC and BAS diets (*P* < 0.05). Plasma isoleucine (**Ile**) and leucine (**Leu**) concentrations were greatest in the PEA diet followed by the BAP and BAS diets (*P* < 0.05). Plasma lysine (**Lys**) and threonine (**Thr**) concentrations were greatest in the PEA diet followed by the BAP and CHK diets, and BAC and BAS diets (*P* < 0.05). Plasma Met was greater in the CHK diet than in the BAS diet (*P* < 0.05). Plasma Phe concentrations were greater in the BAS diet than in the PEA diet (*P* < 0.05). Plasma Trp concentrations were greater in the PEA diet than in the CHK, BAC, and BAS diets (*P* < 0.05). Plasma valine (**Val**) concentrations were greatest in the PEA diet followed by the CHK and BAP diets, and BAS diet (*P* < 0.05). Of the plasma dispensable AAs, Ala concentrations were greatest in the BAS diet, followed by the BAC diet, BAP diet, and PEA diet (*P* < 0.05). Plasma asparagine (**Asn**) concentrations were greatest in the PEA diet followed by the BAP diet, and the CHK, BAC, and BAS diets (*P* < 0.05). Plasma Tyr concentrations were greatest in the BAS diet followed by the BAC and CHK diets, and the PEA diet (*P* < 0.05). Plasma glutamine (**Gln**) concentrations were greater in the PEA and BAS diets than in the CHK diet (*P* < 0.05). Plasma serine (**Ser**) concentrations were greater in the BAS diet than in the PEA diet (*P* < 0.05). There was no effect of diet observed for concentrations of plasma Cys (*P = *0.173), GSH (*P* = 0.589), aspartate (**Asp**; *P* = 0.105), cystine (*P *= 0.219), glutamate (**Glu**; *P* = 0.357), proline (**Pro**; *P* = 0.774), or Tau (*P* = 0.273) across all dietary treatments.

For the whole blood indispensable AA, there was a significant effect of diet for Arg and Ile where concentrations were greatest in the PEA diet (*P* < 0.05). Whole blood concentrations of Leu and Val were greater in the PEA diet than in the BAC and BAS diets (*P* < 0.05). Whole blood concentrations of Lys and Thr were greater in the PEA diet than in the BAP, BAC, and BAS diets (*P* < 0.05). There was no effect of diet observed for concentrations of whole blood His (*P* = 0.426), Met (*P* = 0.100), Phe (*P *= 0.163), or Trp (*P* = 0.066) across all dietary treatments. Of the whole blood dispensable AA there was a significant effect of diet for Ala concentrations which were greatest in the BAS diet followed by the BAC, then the BAP, then the CHK, and finally the PEA diet (*P* < 0.05). Whole blood concentrations of Asn were greater in the PEA diet than in the CHK, BAC, BAP, and BAS diets (*P* < 0.05) while whole blood concentrations of Tyr were greater in the BAS diet than in the PEA, CHK, BAP, and BAC diets (*P* < 0.05). Whole blood concentrations of Gln were greater in the BAS diet than in the BAC and BAP diets (*P* < 0.05) and whole blood concentrations of Glu were greater in the PEA diet than in the BAP diet (*P* < 0.05). There was no effect of diet observed for concentrations of whole blood Asp (*P* = 0.546), cystine (*P* = 0.164), glycine (**Gly**; *P* = 0.259), Pro (*P* = 0.569), or Tau (*P* = 0.292) across all dietary treatments.

Additionally, although the null hypothesis was rejected in the *F*-test, no significant differences were observed between diets for plasma Gly and whole blood serine (*P* = 0.015 and *P* = 0.037, respectively) when pairwise comparisons were analyzed using the Tukey–Kramer adjustment.

### Body weight and calorimetry data

Body weight and calorimetry data are presented in Table 4. There was no difference in BW, mBW, REE, or FEE across all diets (*P *> 0.05) which is a requirement to be able to assess the effect of dietary Met on protein synthesis without changes in whole-body energy metabolism. Fasted and fed RQ and carbohydrate oxidation for dogs eating the BAS and BAC diets was greater than the PEA diet (*P* < 0.05). Fat oxidation was greater for dogs eating the PEA diet than the BAS and BAC diets (*P *< 0.05).

**Table 4.** Bodyweight (BW, kg), metabolic bodyweight (mBW, kg), and indirect calorimetry data for adult large breed dogs (*n *= 10) after indicator amino acid oxidation

	Diet^1^						
Item	BAS	BAC	BAP	CHK	PEA	SEM	*P*-value
% Met req.	50	59	59	68	68		
BW, kg	26.0	26.0	26.2	26.0	26.0	0.80	0.320
mBW, kg^2^	11.5	11.5	11.6	11.5	11.5	0.27	0.321
Resting EE, kcal/kg^0.75^	91.5	93.0	99.0	96.2	98.2	6.53	0.224
Fed EE, kcal/kg^0.75^	117.1	116.9	118.7	118.0	116.7	5.11	0.904
Fasted RQ	0.844^a^	0.850^a^	0.819^a,b^	0.826^a,b^	0.802^b^	0.016	0.006
Fed RQ	0.865^a^	0.861^a^	0.832^a,b^	0.842^a,b^	0.817^b^	0.010	0.001
Fat oxidation, g/min/kg BW	0.097^b^	0.101^b^	0.124^a,b^	0.116^a,b^	0.136^a^	0.009	0.001
Carbohydrate oxidation, g/min/kg BW	0.333^a^	0.323^a^	0.269^a,b^	0.289^a,b^	0.232^b^	0.024	0.001

^1^BAS = basal diet containing lamb as the primary protein source providing Met at 50% of the requirement; BAC = blend of the BAS and CHK diets providing Met at 59% of the requirement; BAP = blend of the BAS and PEA diets providing Met at 59% of the requirement; CHK = blend of the CHK and BAS diets with chicken and lamb as the primary protein sources providing Met at 68% of the requirement; PEA = PEA diet containing peas and lamb as the primary protein sources providing Met at 68% of the requirement.

^2^mBW = BW^0.75^.

## Discussion

To the author’s knowledge, this is the first study attempting to apply the IAAO technique to determine the MA of the limiting AA in ingredients or compounded foods fed to dogs. In order to determine the MA of the limiting AA in ingredients, the reference protein must show a lower level of oxidation of the AA isotope tracer (i.e., C^13^-Phe) indicating greater protein synthesis and bioavailability of the reference protein compared to the test protein (Littell et al., 1997; Moehn et al., 2005). This allows for the MA of the limiting AA in an ingredient to be calculated in relation to the reference protein (Littell et al., 1997; Moehn et al., 2005). In human and pig studies, reference diets based largely on supplying crystalline AAs are used as these are considered to be completely digestible and bioavailable (Moehn et al., 2005; Fakiha et al., 2020). As the CM reference protein had a greater level of oxidation than peas, we were unable to determine the true MA of Met in peas which was 69% greater than CM. Thus, CM was an unsuitable reference protein to determine protein quality in extruded diets intended for dogs.

Chicken meal was chosen as the reference protein because it is a palatable, well-researched protein source that could be easily extruded into kibble to determine the MA of AAs in ingredients provided in this format. However, CM was an inappropriate reference protein due to several factors. First, production of CM through the rendering process utilizes high heat to cook the raw chicken material and separate its fat and water content, resulting in AA losses. For example, Oba et al. (2019) reported that CM had a lower standardized ileal digestibility for Met than retorted, steamed, or raw chicken. Second the extrusion process typically reduces bioavailability of AAs in animal proteins due to heat damage (e.g., Maillard reaction products and Lys) and improves it in plant proteins by deactivating anti-nutritional factors such as phytates (Cargo-Froom et al., 2023b; Geary et al., 2023). It is important to note that MA is different from digestibility in that MA accounts for the portion of a dietary AA that is not only digestible but is also available for protein synthesis (i.e., incorporation into protein; Moehn et al., 2005). Unfortunately, the exact processing parameters used for rendering the CM were proprietary and therefore unavailable to report. Compared to CM, the peas used in this study were only ground and dried prior to extrusion. Therefore, differences in how these raw ingredients were processed would likely negatively impact the portion of Met in these ingredients that was both digestible and bioavailable to different degrees. In general, during heat treatment, Met can be oxidized to Met sulfoxide and Met sulfone, where Met sulfoxide may be reduced back to Met by Met sulfoxide reductase making it available to the animal, however, Met sulfone remains completely unavailable (Rutherford & Moughan, 2008). Current Association of Official Analytical Chemists (**AOAC**) methods for Met determination in food typically call for acid hydrolysis with a performic acid step which will convert all available Met to Met sulfone, leading to a potential overestimation of Met content in ingredients with high heat damage (AOAC, 2005; Rutherford & Moughan, 2008). Therefore, a higher amount of Met sulfone may have been present in CM than peas leading to a reduction in MA of Met in CM. Analyzing the portion of Met, Met sulfoxide, Met sulfone, and presence of cross-linked AAs in each diet and the inherent ingredients was outside the scope of this study but could be considered in the future to better understand how their presence may impact bioavailability of Met in vivo.

Additionally, while poultry proteins have been extensively researched in terms of their AA composition and digestibility and have been used in dog food formulations for decades, they are inherently variable (Donadelli et al., 2019; Oba et al., 2019). It has been widely established that animal proteins have a greater variability in nutrient contents and digestibility compared to plant proteins (Johnson et al., 1998; Bednar et al, 2000; Reilly et al., 2020). For example, apparent ileal AA digestibility of Met in cannulated dogs fed beef and bone meal, poultry byproduct meal, poultry meal, meat and bone meal, and lamb meal have been reported to be 84.7, 87.6, 86.5, 92.7, and 84.3%, respectively (Johnson et al., 1998; Bednar et al, 2000). While Reilly et al. (2020) reported the standardized digestibility of Met in dogs for yellow peas, garbanzo beans, and navy bean powder to be 79.1%, 81.9%, and 80.6%, respectively, using cecectomized roosters. This highlights the importance of having an understanding of the variability within and between common protein-containing ingredients utilized in commercial diets intended for dogs, and the importance of conducting regular ingredient analysis in the raw materials and, ideally, in vivo trials using dogs and animal models to understand how animal proteins and AAs are being digested and utilized. Overall, although CM was not an ideal reference protein for the reasons mentioned above, the difference in MA of Met between CM and peas was still able to be compared. Future studies, however, should aim to develop a crystalline AA reference diet for dogs, as it has been done in other species (Moehn et al., 2005), to allow for determination of the MA of the limiting AA in various intact ingredients, rather than just deriving the difference in MA between ingredients as was done in the current study. This type of reference diet was not originally used in this study in order to provide all dietary components in an extruded format and ensure all other dietary components (other than CM and peas) were processed and metabolized in the same way.

Plasma Met concentrations were below reported reference ranges for healthy adult dogs which was not surprising as Met was provided below the requirement in all diets, however, this effect was not present in whole blood Met (NRC, 2006; Mansilla et al., 2020). This indicates that the graded levels of Met provided as a percent of the Met requirement for Labrador Retrievers (50%, 59%, and 68%, respectively; Mansilla et al., 2020) should have been expanded further below the requirement which may have allowed for determination of differences in whole blood Met concentrations. Interestingly, while plasma Met concentrations were greater in the CHK diet than in the BAS diet, plasma concentrations of Hcys were greater in the PEA diet than in the BAS diet. The concentration of other compounds needed to maintain methylation status, such as choline, folate, and vitamin B12, were similar across all diets and above the recommendations of adult dogs at maintenance (AAFCO, 2023). Therefore, this could suggest that while more Met was present in blood plasma in dogs consuming the CHK diet, more of it was actually utilized in the Met cycle during transmethylation and converted to Hcys (Griffith, 1987) in dogs consuming the PEA diet. This supports the findings in this study where the Met in peas was more bioavailable than in CM. However, elevated blood Hcys (hyperhomocyesteinemia) is often associated with an increased risk of cardiovascular disease and congestive heart failure in humans (Stanger et al., 2003; Refsum et al., 2004). Of recent concern has been the purported increase in reported cases of nutritionally mediated DCM. Dilated cardiomyopathy is the second most common heart disease in dogs differentiated by the enlargement of the left ventricle and reduced contractility of the heart (Dukes-McEwan et al., 2003). Currently, it is unknown what has led to the increased prevalence of nutritionally mediated reported DCM in dogs, but inadequate provision of sulfur-containing AAs Met and Cys from legume ingredients is considered a dominant theory (Mansilla et al., 2019). Rossi et al. (2008) found dogs with existing heart disease (including dogs with DCM) had elevated blood Hcys compared to healthy control dogs (mean Hcys concentration of 10.21 and 5.72 nmol/mL for dogs with heart disease and healthy dogs, respectively). In the current study, dogs consuming the BAS diet and PEA diets had Hcys concentrations 8% and 27% greater than the healthy dog group found by Rossi et al. (2008). However, while dogs consuming the PEA diet had greater plasma Hcys concentrations compared to those consuming the BAS diet, Hcys levels were still 31% less than those diagnosed with heart disease as reported by Rossi et al. (2008). Overall, this suggests that the Met in peas was more metabolically available than CM. Additionally, dogs consuming diets containing a high-inclusion level of peas (44% as-fed) in conjunction with proteins known to have poor Met bioavailability (i.e., lamb) that result in greater plasma Hcys need to be further investigated over time with relevant cardiac measures in order to determine how these diets may impact cardiac function long term.

Although many differences were found in all other plasma and whole blood AA concentrations between dietary treatments, they were generally reflective of the differences in the provision of those AAs in the test diets after blending and supplementation, where the PEA diet contained the highest concentrations of most AAs, followed by the BAP, CHK, BAC, and BAS diets, respectively. For the AAs that were supplemented as free crystalline AAs in the most variable amounts (Phe, Tyr, and Ala), these were generally present in greater concentrations in both plasma and whole blood (e.g., free Phe supplementation in the BAS diet was nearly 40% greater than in the PEA diet). This is expected as crystalline AAs are estimated to be 100% bioavailable and readily absorbed into the systemic circulation (Chung and Baker, 1992). Supplementation of Cys across all diets was similar so no differences in plasma Cys were detected which was expected. The concentration of plasma Trp was both reflective of the amount in the diet, with the PEA diet containing the greatest amount, and minutely by the provision of Trp as free AA. However, supplementation of Trp across the BAS, BAC, and BAP diets was minimal overall which expectedly resulted in no differences in whole blood Trp. It is important to note that other than ensuring Met was provided by both CM and peas at precise graded levels below the requirement, and Cys, Phe, and Tyr provisions were matched across all diets, there was no set upper limit of the provision of all other dietary AAs, only that they were provided above 120% of the AAFCO (2023) requirement of adult dogs at maintenance. The AA requirements (recommended allowances) set by AAFCO (2023) were used as opposed to the recommended allowance set by the NRC (2006) for adult dogs at maintenance in order to validate this method as AAFCO AA recommendations are generally inflated to cover for variability in AA digestibility in ingredients and processing conditions. Overall, concentrations of plasma and whole blood AAs across dietary treatments were generally within reported ranges found in healthy adult dogs at maintenance (apart from Ala which was above, and Met and GSH which were below the reported range; Delaney et al., 2003; NRC, 2006; Beloshapka et al., 2018; Banton et al., 2021).

Bodyweight and fasted and fed EE remained similar across all treatments, suggesting that different dietary intake of Met from both CM and peas did not affect energy metabolism and that all dogs were fed to their individual energy requirements, as is required for design principles in MA studies on animals at maintenance (Moehn et al., 2005; Shoveller et al., 2017). Dogs fed the PEA diet had lower fasted and fed RQ, lower carbohydrate oxidation, and greater fat oxidation compared to dogs consuming the BAS and BAC diets which is likely due to the following factors. In terms of diet composition, although diets were isoenergetic, the BAS and BAC diets had 30 and 18% greater calculated NFE, respectively, and 30% and 26% less crude fat, respectively, compared to the PEA diet. There is a dearth of research that explores how different diet macronutrient compositions impact RQ in dogs. In humans, however, Goldenshluger et al. (2021) reported that consumption of diets with lower carbohydrates and higher fat concentrations showed lower RQs compared to diets with lower fat concentrations, which was also observed herein. Additionally, the supplementation of Ala to make all diets isonitrogenous, particularly in the BAS and BAC diets (at nearly 10 and 8% DM, respectively), likely resulted in a further increase in glycolysis rate and overall carbohydrate oxidation in dogs consuming these diets. Alanine is a key gluconeogenic AA and readily converted to glucose, peaking at 30 to 60 min post intravenous dosing in humans (Felig, 1973). As Ala was given orally as free AA in solution and fed-state blood samples were taken within 30 min of the last meal, it is reasonable that Ala supplementation at such a high concentration remained in the systemic circulation by the time blood was sampled, resulting in greater RQ and carbohydrate oxidation in dogs consuming the BAS and BAC diets. Although the application of the IAAO technique to determine bioavailability of limiting AAs in ingredients requires precise diet design as discussed previously, other dietary factors, such as fiber and macronutrient balance are considered inherent to the test ingredients themselves (Moehn et al., 2005). Therefore, it is reasonable that the different macronutrient compositions of the test diets and Ala supplementation resulted in a shift in macronutrient oxidation.

## Conclusions and Implications

The data presented above suggests that the diet design and execution of the IAAO technique to determine MA of Met in both CM and peas was successful in creating a linear response in oxidation to graded intakes of protein-bound Met from these ingredients. In this study, it was determined that the MA of Met in peas was 69% greater than the MA of Met in the CM used herein. Due to the lack of a reference diet designed to supply Met in its 100% bioavailable form, the specific MA of Met in peas could not be determined. Future research should explore the development of a reference diet that provides the test limiting AA in its 100% bioavailable form, while still resembling the nature of an extruded diet to consider, somehow, the complex matrix of pet food and the effects of thermal and mechanical inputs on nutrient interaction and losses.

## Abbreviations

AA: amino acid
AAFCO: Association of American Feed Control Officials
AOAC: Association of Official Analytical Chemists
APE: atom percent excess
BW: body weight
CM: chicken meal
DCM: dilated cardiomyopathy
DM: dry matter
FEE: fed energy expenditure
IAAO: indicator amino acid oxidation
MA: metabolic availability
mBW: metabolic BW
NaOH: sodium hydroxide
REE: resting energy expenditure
RQ: respiratory quotient
SAA: sulfur amino acid
UPLC: ultra performance liquid chromatography
VCO_2_: volume of expired carbon dioxide
VO_2_: volume of expired oxygen
